# Across-Experiment Transcriptomics of Sheep Rumen Identifies Expression of Lipid/Oxo-Acid Metabolism and Muscle Cell Junction Genes Associated With Variation in Methane-Related Phenotypes

**DOI:** 10.3389/fgene.2018.00330

**Published:** 2018-08-20

**Authors:** Ruidong Xiang, Jody McNally, Jude Bond, David Tucker, Margaret Cameron, Alistair J. Donaldson, Katie L. Austin, Suzanne Rowe, Arjan Jonker, Cesar S. Pinares-Patino, John C. McEwan, Phil E. Vercoe, V. H. Oddy, Brian P. Dalrymple

**Affiliations:** ^1^CSIRO Agriculture & Food, Queensland Bioscience Precinct, St Lucia, QLD, Australia; ^2^Faculty of Veterinary and Agricultural Sciences, The University of Melbourne, Parkville, VIC, Australia; ^3^Agriculture Victoria, AgriBio, Centre for AgriBioscience, Bundoora, VIC, Australia; ^4^F. D. McMaster Laboratory, CSIRO Agriculture & Food, Armidale, NSW, Australia; ^5^NSW Department of Primary Industries, Extensive Livestock Industries Centre, University of New England, Armidale, NSW, Australia; ^6^Invermay Agricultural Centre, AgResearch Limited, Mosgiel, New Zealand; ^7^Grasslands Research Centre, AgResearch Limited, Palmerston North, New Zealand; ^8^New Zealand-Peru Dairy Support Project, MINAGRI, Lima, Peru; ^9^School of Animal Biology, The University of Western Australia, Crawley, WA, Australia; ^10^Institute of Agriculture, The University of Western Australia, Crawley, WA, Australia

**Keywords:** sheep, transcriptomics, rumen mean retention time, methane, oxo-acid metabolism, smooth muscle

## Abstract

Ruminants are significant contributors to the livestock generated component of the greenhouse gas, methane (CH_4_). The CH_4_ is primarily produced by the rumen microbes. Although the composition of the diet and animal intake amount have the largest effect on CH_4_ production and yield (CH_4_ production/dry matter intake, DMI), the host also influences CH_4_ yield. Shorter rumen feed mean retention time (MRT) is associated with higher dry matter intake and lower CH_4_ yield, but the molecular mechanism(s) by which the host affects CH_4_ production remain unclear. We integrated rumen wall transcriptome data and CH_4_ phenotypes from two independent experiments conducted with sheep in Australia (AUS, *n* = 62) and New Zealand (NZ, *n* = 24). The inclusion of the AUS data validated the previously identified clusters and gene sets representing rumen epithelial, metabolic and muscular functions. In addition, the expression of the cell cycle genes as a group was consistently positively correlated with acetate and butyrate concentrations (*p* < 0.05, based on AUS and NZ data together). The expression of a group of metabolic genes showed positive correlations in both AUS and NZ datasets with CH_4_ production (*p* < 0.05) and yield (*p* < 0.01). These genes encode key enzymes in the ketone body synthesis pathway and included members of the poorly characterized aldo-keto reductase 1C (*AKR1C*) family. Several *AKR1C* family genes appear to have ruminant specific evolution patterns, supporting their specialized roles in the ruminants. Combining differential gene expression in the rumen wall muscle of the shortest and longest MRT AUS animals (no data available for the NZ animals) with correlation and network analysis, we identified a set of rumen muscle genes involved in cell junctions as potential regulators of MRT, presumably by influencing contraction rates of the smooth muscle component of the rumen wall. Higher rumen expression of these genes, including *SYNPO* (synaptopodin, *p* < 0.01) and *NEXN* (nexilin, *p* < 0.05), was associated with lower CH_4_ yield in both AUS and NZ datasets. Unlike the metabolic genes, the variations in the expression of which may reflect the availability of rumen metabolites, the muscle genes are currently our best candidates for causal genes that influence CH_4_ yield.

## Introduction

The growing global human population is resulting in increased demand for meat and fiber products from livestock. While satisfying human demands, increasing ruminant numbers, including cattle and sheep, have also resulted in emissions of increasing amounts of methane (CH_4_) (Pachauri et al., 2014). The CH_4_ generated by the rumen enteric fermentation contributes approximately 13.8–26.2% of the global anthropogenic CH_4_ (Prather et al., 2012; Hill et al., 2016). However, ruminants are the major means of converting low quality/human-inedible cellulosic plant material to high value animal protein (Chalupa, 1978; Wilkinson and Lee, 2017). Also, a recent study in the United States has shown that the complete removal of livestock from the agriculture sector will make the food supply incapable of meeting nutritional requirements (White and Hall, 2017). Increasing the production of high quality meat while reducing CH_4_ produced by herbivores has been one of the major aims of international collaborations in livestock research (Scollan et al., 2011). Additionally, mitigation of ruminant CH_4_ production offers the opportunity to increase feed energy utilization in the host animal while reducing environmental impact (Pickering et al., 2015).

Significant international research efforts have been directed to find ways to mitigate ruminant CH_4_ production without negatively impacting animal production. Methanogen inhibitors added to the feeds have been shown to have CH_4_ reducing effects in dairy cows without negative effects on production (Duin et al., 2016). However, such approaches may only be practical in intensive production systems with total mixed rations, primarily intensive dairy-farms and feedlots. In more extensive production systems, such as grazing dairy and beef cattle and sheep, alternative approaches may be required (Wedderburn-Bisshop et al., 2015).

One alternative way to reduce livestock CH_4_ emission is to select animals with low CH_4_ yield. Although the heritability of CH_4_ yield appears to be low in sheep (0.13) (Pinares-Patino et al., 2013; Goopy et al., 2016), host genetic effects on enteric CH_4_ have been demonstrated in ruminants (Pinares-Patino et al., 2013; Goopy et al., 2016; Roehe et al., 2016). It has been reported in a number of independent experiments that rumen size, rumen feed particle mean retention time (MRT) and CH_4_ yield (CH_4_ production/dry matter intake, DMI, kg/day) are correlated in sheep (Pinares-Patiño et al., 2003, 2011; Elmes et al., 2014; Goopy et al., 2014; Bond et al., 2017). A mechanistic rumen simulation study predicted similar relationships (Huhtanen et al., 2016). The contraction frequency and force of the muscles in the reticulo-rumen wall and reticulo-omasal orifice contribute to the control of digesta MRT in the rumen (Bueno and Ruckebusch, 1974; Okine et al., 1989; Okine and Mathison, 1991).

Our previous study (Xiang et al., 2016a) identified some of the mechanisms by which the rumen wall interacts with diet which eventually leads to CH_4_ production. Unfortunately, gene expression correlation, and gene expression with phenotype correlation, studies are inherently noisy, especially when small numbers of animals are involved. Due to the small number of experimental animals, we were not able to identify significant rumen wall pathways associated with net CH_4_ production and yield (Xiang et al., 2016a). However, integration of data from independent studies (including those with different environmental conditions and different populations) can mitigate the impact of noise. In the present study, we generated a dataset of sheep rumen wall transcriptome from an experiment where CH_4_ phenotypes have been studied (Bond et al., 2017). These transcriptome data (Australia, AUS) (available with the accession number GSE81847, see the Section “Availability of Data and Material”) were jointly analyzed with a previously published set of rumen wall transcriptome data (available with the accession number PRJNA313132) generated from New Zealand (NZ) sheep where CH_4_ phenotypes were also measured (Xiang et al., 2016a). By integrating data from the different experiments, we identified consistent gene expression clusters representing major rumen functions, including potential affectors of MRT.

## Results and Discussion

### Differences Between the Two Experiments

In this study we integrated and analyzed a sheep (*n* = 62) full thickness rumen wall transcriptome data set generated from an experiment conducted in the AUS dataset (Bond et al., 2017), with the previously described dataset from an independent experiment conducted in NZ (*n* = 24) (Xiang et al., 2016a), the NZ dataset (**Table 1**). The two experiments had very different designs (**Table 1**), although both were directed at understanding the genetic background of CH_4_ production by sheep. The NZ experiment was conducted with younger sheep (up to 1 year) of different breed composition than in the AUS experiment and included four levels of dietary perturbations. In the AUS experiment, older animals (> 3 years) were randomly assigned to four blocks (test periods) for phenotyping and were fed the same diet at 1.5 × maintenance (**Table 1**).

**Table 1:** Summary of sheep methane measurement experiments in Australia (Bond et al., 2017) and New Zealand (Xiang et al., 2016a).

Locale	Breeds	Sex	Age (mo.)	Feeding regime	Forage type	DE^a^	Rumen sampling	Block	Animals	Diet conditions
AUS	Merino	♀	∼30	08:00 and 16:00	lucerne: oat chaff (50:50)	12.1	10:00–11:00	1	16	1.5 × maintenance
								2	16	
								3	14	
								4	16	
NZ	Composite^b^	♀	∼9	08:30 and 15:30	cut rye grass pasture	11.6 poor 12.2 good	before morning feed	na	6	good+1.0 × maintenance
									6	poor+1.0 × maintenance
									6	good+1.5 × maintenance
									6	poor+1.5 × maintenance


*^*a*^Digestible energy (MJ/kg DMI) was calculated by converting metabolizable energy of the AUS experiment. The rumen sampling in the AUS experiment took place 2–3 h post feeding (Bond et al., 2017). For the NZ experiment, digestible energy was calculated from gross energy and *in vitro* digestibility (see methods). ^*b*^See details in Pinares-Patino et al. (2013).*

Comparing the phenotyping results for the two experiments, the CH_4_ parameters were significantly different, with higher CH_4_ production, but lower CH_4_ yield for the AUS animals (**Supplementary Table S1**). A significant negative correlation between CH_4_ yield and DMI was observed in AUS experiment (correlation coefficient *r* = -0.33, *p* < 0.01). The relationship between CH_4_ yield and DMI in the NZ experiment was negative (*r* = -0.34) but insignificant (*p* = 0.11). Previously, negative relationships between CH_4_ yield and intake in sheep have been reported (Blaxter and Clapperton, 1965; Swainson et al., 2015). No significant relationships existed between CH_4_ production and CH_4_ yield in either dataset (**Supplementary Table S1**), or when the datasets were combined (**Supplementary Figure S1**). The NZ animals were fed fresh ryegrass-based forage, whereas the AUS animals were fed chaffed mixed lucerne and cereal hay. Feeding different diets will result in different methane yields due to differences in feed composition or digestibility (Archimède et al., 2011; Doreau et al., 2011; Kennedy and Charmley, 2012). The lower digestible energy (DE) in the feed of AUS experiment (**Table 1**) may have led to less substrate for fermentation, resulting in lower CH_4_ yield, but higher CH_4_ production, compared to the NZ experiment. In addition, the age of ruminants can significantly change the compositions of rumen bacteria and the methanogens that produce CH_4_ (Liu et al., 2017)_._ The concentrations of principal short chain fatty acids (SCFA), including acetate, butyrate and propionate, were significantly higher for the AUS than NZ animals (**Supplementary Table S1**). Significant correlations between DMI and SCFA concentrations were only found in the NZ experiment (**Supplementary Table S2**). These differences may reflect the different sampling times in the AUS and NZ animals relative to the feeding of the animals (**Table 1**). However, in both groups of animals, the concentrations of the three principal SCFAs, acetate, butyrate, and propionate were generally highly correlated with each other (**Supplementary Table S2**).

### Cross-Experiment Rumen Transcriptomic Network

**FIGURE 1:**
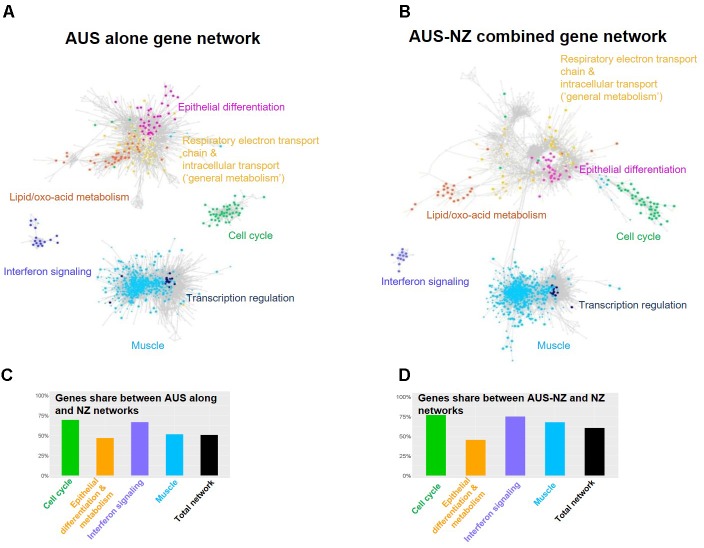
Relationships between transcriptomes from the AUS and NZ animals. Transcriptome networks based on AUS alone **(A)**, and AUS-NZ combined **(B)** data, with genes included in the major clusters of the networks and identified in the previously generated NZ network (Xiang et al., 2016a) indicated (**Supplementary Figure S3**). The networks were built based on correlation of voom normalized gene expression (Law et al., 2014). For the AUS-NZ combined data **(B)**, quantile normalization was used to remove non-biological variations (Bolstad et al., 2003). **(C)** The percentage of genes in the AUS network and in each major cluster of the AUS network that were included in the NZ network and major gene clusters. **(D)** The percentage of genes in the AUS-NZ combined network and in each major cluster of the AUS-NZ combined network that were included in the NZ network and major gene clusters.

Principal component analysis implemented in DESeq2 (Love et al., 2014) using the AUS and NZ count data along with RNA seq data generated by The Roslin Institute in previous studies (Jiang et al., 2014; Xiang et al., 2016b) demonstrated that the AUS and NZ rumen transcriptome data were tightly clustered (**Supplementary Figure S2**). We integrated the AUS and NZ rumen wall transcriptome data and compared the relationships between rumen gene expression clusters and CH_4_-related phenotypes between the two experiments. Given the significant differences between the two experiments, such an integrative analysis provides a strong negative control for unreproducible gene-to-phenotype correlations. After filtering out transcripts with low levels of expression [count per million (CPM) < = 3 in all individuals], the AUS (**Figure 1A**) and AUS and NZ combined (AUS-NZ) (**Figure 1B**) background corrected transcriptomic networks were built using the same parameters as the previously described for the NZ dataset (Xiang et al., 2016a). The Glay community clustering plugin in Cytoscape was used to identify gene sub-clusters based on gene-gene correlation pairs with correlation *r* > 0.8 and satisfying the information content criteria imposed by PCIT (Reverter and Chan, 2008). The gene network overlap for the two independent experiments (AUS and NZ) was highly significant (hypergeometric hP < 1.0e-100). More than 50% of genes in both the AUS and AUS-NZ combined networks were present in the previously constructed NZ transcriptomic network (**Figures 1C,D** and **Supplementary Figures S2**, **S3**). The two major gene clusters identified in the NZ data, epithelium and muscle layer-based genes (Xiang et al., 2016a) were also present in the AUS (**Figure 1A**) and the AUS-NZ networks (**Figure 1B**). Based on the previous network biological pathway annotation (Xiang et al., 2016a) and their presence in both the AUS (**Figure 1A**) and the AUS-NZ networks (**Figure 1B**), we defined five gene sub-clusters, which we will focus on in this study. The epithelium gene cluster was divided into four sub-clusters: “cell cycle,” “epithelial differentiation,” “lipid/oxo-acid metabolism,” and “general metabolism.” The muscle gene cluster was defined based on the inclusion of genes in both the AUS and AUS-NZ networks without further subdivision. Most of the major gene sub-clusters had more than 50% of genes in common between the AUS and NZ experiments (**Figures 1C,D**). The list of genes shared between the AUS and NZ networks with their sub-cluster and enrichment information can be found in **Supplementary Table S3**.

**FIGURE 2:**
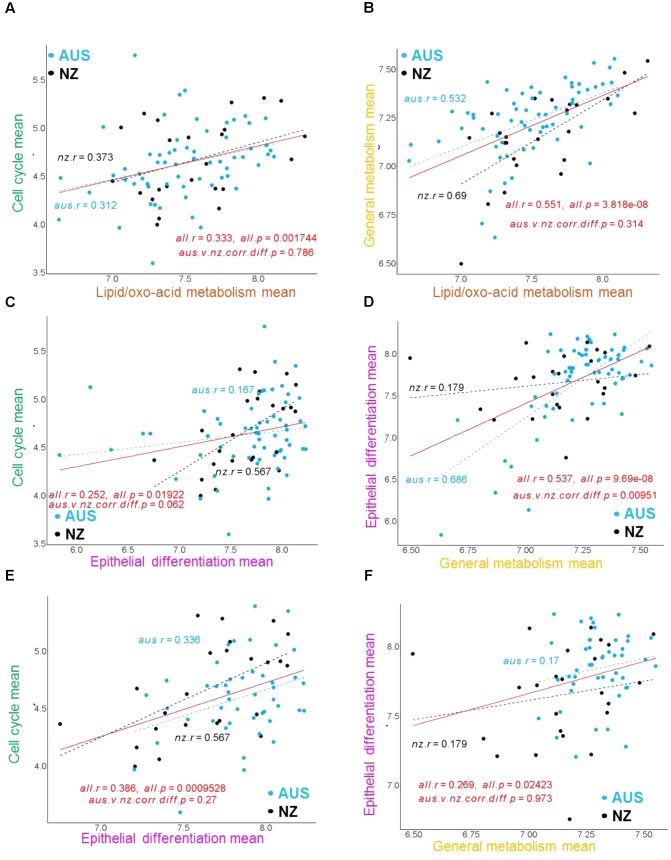
Examples of correlation regressions between the mean expression of major gene clusters in the AUS and NZ datasets. The correlations and significance of between-experiment difference in correlations for mean gene cluster expressions are indicated for each analysis on the relevant panel. The mean expression of the set of genes in each gene cluster for each animal was calculated from the voom (Law et al., 2014) normalized gene expression values for each gene in each animal generated by normalizing the AUS and NZ datasets together. Quantile normalization was used to remove non-biological variations (Bolstad et al., 2003). **(A–D)** Correlation regressions with all AUS animals included in the analysis. **(E,F)** Correlation regressions without the AUS block 1 animals.

Previously, genes from the annotated sub-clusters with the epithelial origin including “cell cycle,” “epithelial differentiation,” “general metabolism,” and “lipid/oxo-acid metabolism” (**Supplementary Table S3**) showed correlations with CH_4_ phenotypes (Xiang et al., 2016a). In the present study, we examined the relationships between these gene sub-clusters in the AUS and NZ datasets (**Figure 2**). The mean expression of the set of genes included in each of these four annotated sub-clusters for each of the individual AUS and NZ animals (calculated from the jointly normalized AUS-NZ dataset) was used to represent the expression of the respective sub-clusters in subsequent analyses (**Figure 2**). In agreement with the networks based on individual genes (**Figure 1**), there were consistently positive relationships between the mean expression levels of the cell cycle genes, lipid/oxo-acid metabolism, general metabolism, and epithelial differentiation gene sets in both datasets (**Figures 2A–D**). This suggests generally coordinated cell proliferation and metabolism in the rumen epithelial system.

Differences in gene set relationships between the experiments were also observed. The overall significant positive relationship of mean expression between cell cycle genes and epithelial differentiation genes was largely driven by the strong relationship in the NZ dataset (**Figure 2C**). At the network level, the cell cycle genes in the NZ dataset (Xiang et al., 2016a) (**Supplementary Figure S3**) were well connected with the epithelial differentiation genes, while in the AUS dataset the cell cycle genes stood out as an independent cluster (**Figure 1A**). Conversely, the overall significant positive relationship of mean expression between the epithelial differentiation genes and general metabolism genes was driven by the strong relationship in the AUS dataset (**Figure 2D**). At the network level, previously separated epithelial differentiation and general metabolic genes in the NZ dataset (Xiang et al., 2016a) were well connected in the AUS dataset (**Figure 1A**). However, further analysis identified that the expression level of the epithelial differentiation genes was significantly lower in animals of test block 1 in the AUS experiment than for animals in blocks 2–4 (**Supplementary Figure S4**). The reasons for this observation are unknown. There are no significant differences in CH_4_ production between the blocks (Bond et al., 2017). While CH_4_ yield had a difference (*p* < 0.022) between blocks, this was driven by the 4th block (CH_4_ yield g/d/DMI 20.2 ± 0.66), compared to the other three blocks (18.3 ± 0.66 for block 1, 18.7 ± 0.66 for block 2, and 19.0 ± 0.66 for block 3) (Bond et al., 2017). Exclusion of the animals from block 1 of the AUS experiment from the analysis resulted in similar relationships between the epithelial differentiation and general metabolic genes in the AUS and NZ animals (**Figures 2E,F**). As the effect is limited on the other gene sets, the impact on the CH_4_ phenotypes is small, and it is unclear whether the differences result from true biological variation or technical issues, the data from animals of block 1 were included in the analyses reported in this paper.

In this comparison of two datasets from completely independent animals derived from full depth rumen wall samples from sheep, the basic organizational structure of the gene expression network was conserved. This suggests that the network reflects the fundamental biological activities of the rumen wall and provides a framework for the interpretation of studies of gene expression in the rumen wall.

### Genes Encoding the Ketone-Body Synthesis Pathway

**FIGURE 3:**
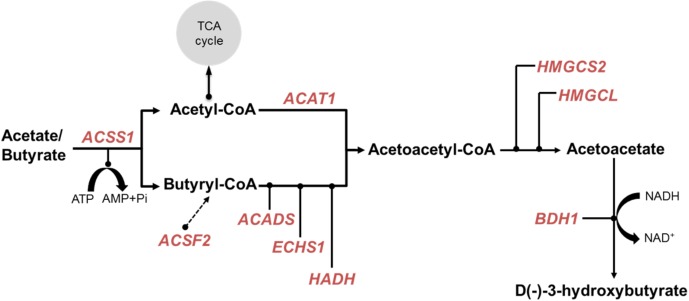
Simplified ketone body metabolic pathway. Key enzymes were based on the encoding genes which were present in both AUS and NZ (Xiang et al., 2016a) metabolism gene networks. These presented genes were indicated by red text. Dashed arrow represents a hypothesized contribution of *ACSF2* to butyryl-CoA synthesis.

The ketone-body synthesis pathway in the rumen epithelium is involved in the acquisition of energy by the ruminants (Lane et al., 2002) and the concentration of circulating β-hydroxybutyrate is the higher in ruminants compared to human and mice (Robinson and Williamson, 1980). Our previous analysis has shown that many of the genes encoding the enzymes of the ketone-body synthesis pathway are preferentially expressed in the rumen (Xiang et al., 2016b). This is supported by a recent comprehensive survey of gene expression in sheep (Clark et al., 2017) (**Supplementary Table S4**). In the present study, the metabolism gene cluster contained: *ACAT1* (acetyl-CoA acetyltransferase 1), *ACADS* (acyl-CoA dehydrogenase short chain), *ECHS1* (enoyl-CoA hydratase, short chain 1), *HMGCS2* (3-hydroxy-3-methylglutaryl-CoA synthase 2), *HMGCL* (3-hydroxymethyl-3-methylglutaryl-CoA lyase), and *BDH1* (3-hydroxybutyrate dehydrogenase 1) (**Figure 3**). *ACSS1* (acyl-CoA synthetase short chain family member 1) presumed to encode the enzyme converting acetate and butyrate to their –CoA derivatives was present in the muscle cluster, possibly reflecting a more general role of ACSS1 in metabolism than the other enzymes. Of the candidate genes for enzymes catalyzing the conversion of L+3-hydroxy-butyryl-CoA to Acetoacetyl-CoA (**Figure 3**), only HADH (hydroxyacyl-CoA dehydrogenase), and neither EHHADH (enoyl-CoA hydratase and 3-hydroxyacyl CoA dehydrogenase) nor HADHA (hydroxyacyl-CoA dehydrogenase trifunctional multienzyme complex subunit alpha), were present in the metabolism cluster. *OXCT1* (3-oxoacid CoA-transferase 1), which encodes an enzyme providing an alternate pathway from acetoacetyl-CoA to acetoacetate, was also not present in the network. Therefore, a simplified ketone-body pathway can be drawn for the sheep rumen (and likely also for other ruminants) (**Figure 3**). Interestingly, *ACSF2* (acyl-CoA synthetase family member 2) from the metabolism cluster is also preferentially expressed in rumen (**Supplementary Table S4**), but was not included in the previously proposed ketone body metabolism pathways (Xiang et al., 2016b). Given that the rumen epithelium can also convert long-chain and non-esterified fatty acids from the blood circulation into ketone bodies (Jackson et al., 1964; Taylor and Jackson, 1968), the *ACSF2* encoded enzyme may have a role in directing other, longer chain lipids into the ketone body pathway, and thus, is a potential candidate for a butyryl-CoA synthetase for future experimental follow-ups.

### Gene Sets Associated With CH_4_ Production

**FIGURE 4:**
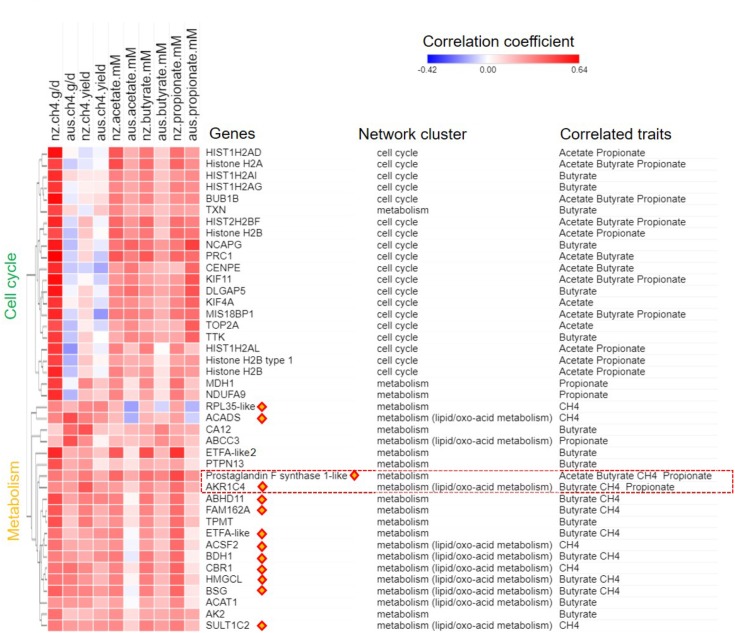
Gene-phenotype correlation heat map for CH_4_ traits and short chain fatty acids (SCFAs) concentrations. Genes identified as associated with CH_4_ production (g/day) or yield (g/day/dry matter intake, kg/d; **Figure 2**), or correlated with individual SCFA concentrations (mM) are shown. Values for the heatmap are the correlation coefficients with the respective phenotype in the AUS and NZ datasets. Genes are annotated for names, network origin (**Figure 1A**) and for which trait they were identified as consistently correlated with. The correlation coefficient heat map was clustered based on Euclidean distance. Orange diamonds highlight metabolic genes always showing correlations with CH_4_ production and yield (**Figure 2**). The red frame highlights poorly annotated *AKR1C* family members with probable ruminant specific evolution (**Figure 5**).

A joint analysis of the AUS and NZ data was undertaken to identify genes with expression consistently correlated with CH_4_ production (g/d) in both datasets. Our step-wise joint analyses (see the Section “Materials and Methods”) differ from traditional gene selection approaches where hard cutoffs such as *p*-values are used. Borrowing the idea of using soft cutoffs such as implemented in PCIT (Reverter and Chan, 2008), we started from the separate sets of genes with CPM value > 1 in all samples in the AUS (16,011 genes) or the NZ (15,572 genes) datasets. For each phenotype, genes with gene-phenotype correlation within upper 1/3 quantile (top 33%) were chosen to perform PCIT analysis to construct small gene networks independently from the AUS and NZ datasets. The unique gene lists from the small AUS and NZ networks were merged and we also further filtered out genes with absolute correlation values of less than 0.15 in both datasets, and that did not show the same correlation sign in the AUS and NZ correlation analyses. This approach considered both gene-phenotype and gene-gene relationships and the consistency of correlations between datasets. While this analysis was exhaustive, it did not perform any significance tests, but rather focused on soft cutoffs of correlation ranking and inclusion in different datasets, which has been shown to have power for finding biologically informative gene sets correlated with complex traits (Guo et al., 2014; Henderson et al., 2017). All identified gene set members associated with respective phenotype are shown together with their correlation coefficients with the traits in both the AUS and NZ datasets (**Figure 4**). The IDs of all identified genes and their correlation data are shown in **Supplementary Table S5**.

The cell cycle genes did not show consistent correlations with CH_4_ production or yield in the two datasets. In the NZ dataset, the expression of the cell cycle genes in the full thickness rumen wall samples had average *r* = 0.54 with CH_4_ production (**Figure 4** and **Supplementary Table S5**). In the AUS dataset, however, the same genes showed average *r* = -0.06 with CH_4_ production (**Figure 4** and **Supplementary Table S5**). Thus, there does not appear to be a consistent relationship between the expression of cell cycle genes in the rumen of sheep and CH_4_ production. While it is probable that the original result with the NZ animals was a spurious correlation, it is also possible that differences in experimental design and in particular the feed composition and feeding levels in the two experiments (**Table 1**) has contributed to real differences in the relationship.

**FIGURE 5:**
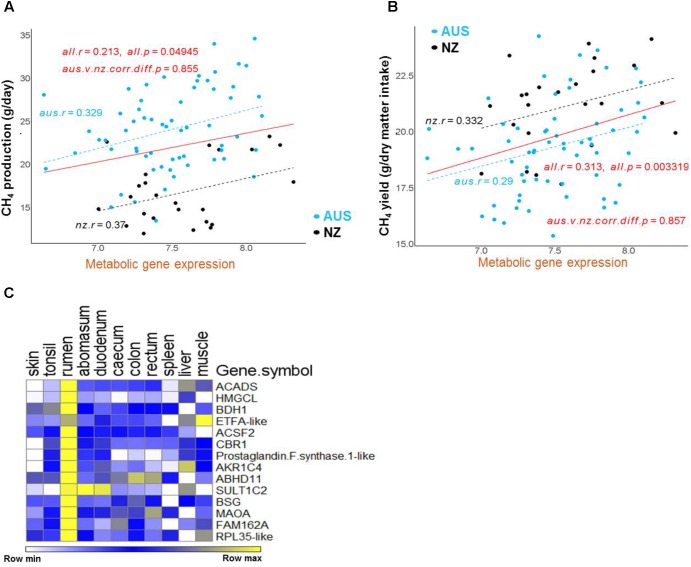
Regressions of CH_4_ production and yield on the expression of the metabolic gene set in the AUS and NZ experiments. **(A,B)** Correlation regressions between the mean expression of the metabolic gene set and CH_4_ production **(A)** or CH_4_ yield **(B)** in each animal. The mean expression of the set of genes in each gene cluster for each animal was calculated from the normalized gene expression values for each gene in each animal generated by normalizing the AUS and NZ datasets together. The significance of between-experiment difference of correlations of mean gene cluster expressions is indicated in the relevant panel. **(C)** The heatmap of the metabolic gene set expression using data from the sheep RNA-seq atlas (Xiang et al., 2016b).

The genes from the metabolic cluster had the most consistent correlations with CH_4_ production across the two experiments (**Figures 4**, **5A**). These genes included many members of the lipid/oxo-acid gene set (see above), in particular three genes encoding enzymes in the ketone body synthesis pathway (**Figure 3** and **Supplementary Table S4**), and labeled in orange diamonds on **Figure 4**). When regressing the CH_4_ production on the mean expression of this set of metabolic genes using both the NZ and AUS animals, consistent positive relationships (no significant difference in the slopes) were observed (**Figure 5A**). The only evident difference between the NZ and AUS regressions were the different intercepts, which were consistent with the significant differences in CH_4_ production between the animals in the two experiments. Since variation in CH_4_ production is mainly determined by DMI (Ramin and Huhtanen, 2013), which was also observed in our study (**Supplementary Table S2**), the intercept differences in two experiments associated with CH_4_ production is most likely to be attributable to the different intakes of the animals in the two experiments.

### Gene Sets Associated With CH_4_ Yield

In the analysis of the NZ dataset, the expression of a large number of the lipid/oxo-acid metabolism genes (73 gene members) was weakly positively correlated with CH_4_ yield (Xiang et al., 2016a). In order to validate these findings, a joint analysis of the AUS and NZ data was undertaken and a relatively small set of genes (*n* = 14) were identified that consistently correlated with CH_4_ yield in both datasets (**Figure 4**). Of these 14 genes, 8 were in the lipid/oxo-acid metabolism gene cluster. This overlap was small but significantly more than expected by random chance (hP < 5.4e-16). The other 6 were in rumen epithelium “general metabolism” gene cluster (742 gene members) (Xiang et al., 2016a).

Furthermore, 14 of above identified metabolic genes (**Table 2**), whose expression levels were correlated with CH_4_ yield, also had positive correlations with CH_4_ production (labeled with orange diamonds on **Figure 4**). The regressions of the CH_4_ yield on the expression of the set of 14 metabolic genes common to the two experiments are shown in **Figure 5B**, where there was no significant difference in the slopes of the two regression lines. The difference in regression intercepts was again due to the significant difference in CH_4_ yield between the two experiments (see above). Thus, the expression level of the metabolism genes is an indicator of CH_4_ yield or production within, but not between, experiments. This suggests that the level of expression of the metabolism genes is affected by the level of an intermediate in the process from feed to CH_4_ involving the dynamic interactions between the host factors, the rumen microbes and feed compositions and that the relationship between this intermediate and CH_4_ production is consistent within, but not between experiments.

**Table 2:** Annotation of metabolic genes always correlated with CH_4_ production and yield in Australian and New Zealand datasets.

ID	Symbol	Function	Pathway	Subcellular location^a^
ENSOARG00000013118	*ACADS*^b^	acyl-CoA dehydrogenase	ketone body synthesis	mitochondria
ENSOARG00000007033	*HMGCL*^b,c^	3-hydroxymethyl-3-methylglutaryl-CoA lyase	ketone body synthesis	mitochondria
ENSOARG00000020383	*BDH1*^b^	3-hydroxybutyrate dehydrogenase	ketone body synthesis	mitochondria
ENSOARG00000010175	*ETFA-like*	shuttles electrons between primary flavoprotein (acyl-CoA) dehydrogenases and the membrane-bound electron transfer flavoprotein ubiquinone oxidoreductase	ketone body synthesis?	mitochondria
ENSOARG00000004315	*ACSF2*^c^	acyl-CoA synthetase	ketone body synthesis?	mitochondria
ENSOARG00000013700	*CBR1*^c^	carbonyl reductase	arachidonic metabolism?	cytosol
ENSOARG00000010221	Prostaglandin F synthase1-like	aldo-keto reductase	arachidonic metabolism?	–
ENSOARG00000009762	*AKR1C4*^c^	aldo-keto reductase	arachidonic metabolism?	cytosol
ENSOARG00000012026	*ABHD11*^b^	hydrolase	–	mitochondria
ENSOARG00000000097	*SULT1C2*^c^	sulfotransferase	–	plasma membrane
ENSOARG00000008783	*BSG*^c^	immunoglobulin	–	extracellular
ENSOARG00000003052	*MAOA*^b^	catalyzes the oxidative deamination of amines	–	–
ENSOARG00000020132	*FAM162A*	unknown	–	intracellular
ENSOARG00000001203	*RPL35-like*	unknown	–	–


*^*a*^Subcellular location was obtained from Genatlas (Frézal, 1998). ^*b*^Overlap with gene differentially expressed in starter fed versus milk fed calves (Connor et al., 2013). ^*c*^Overlap with genes differentially expressed in starter fed versus milk fed lambs (Wang et al., 2016).*

### Functions of the Genes Associated With Methane Production and Yield

The finding that a set of 14 metabolic genes (**Table 2**) showed positive correlations with both CH_4_ production and CH_4_ yield, which had an insignificant relationship with each other (**Supplementary Table S2**), suggested that they have a unique function in ruminant metabolism. Interestingly, by re-analyzing published data from the transcriptomic study of sheep gastrointestinal tract (Xiang et al., 2016b), we show that all of the 14 metabolic genes had higher expression in the rumen than in all of the other tissues in the gastrointestinal tract, spleen and liver, and all but one of the genes also had high expression in the muscle (**Figure 5C**). Such a tissue expression pattern is also generally consistent with the reported results from the latest sheep transcriptome atlas (Clark et al., 2017) (**Supplementary Table S4**). Three of the 14 genes, including *ACADS*, *HMGCL*, and *BDH1*, encode key enzymes in the ketone body synthesis pathway (Lane et al., 2002) (**Table 2** and **Supplementary Table S4**). Two additional genes encode metabolic enzymes, the function of which is consistent with a role in ketone body synthesis (**Table 2**). Specifically, ETFA-like which may play a role in shuttling electrons between primary flavoprotein dehydrogenases and flavoprotein ubiquinone oxidoreductase. Reduced function of ETFA has been associated with deficiencies in the activity of multiple acyl-CoA dehydrogenases (Chiong et al., 2007), which is part of the ketone body metabolic pathway (**Figure 3**, Lane et al., 2002). Five of the 14 genes (hP < 0.001) were more highly expressed in starter fed than milk fed calves (Connor et al., 2013) and six of the 14 genes (hP < 0.001) were more highly expressed in starter fed than milk fed lambs (Wang et al., 2016) (**Table 2**), consistent with the genes encoding enzymes with a role in the acquisition of energy from solid diets.

**FIGURE 6:**
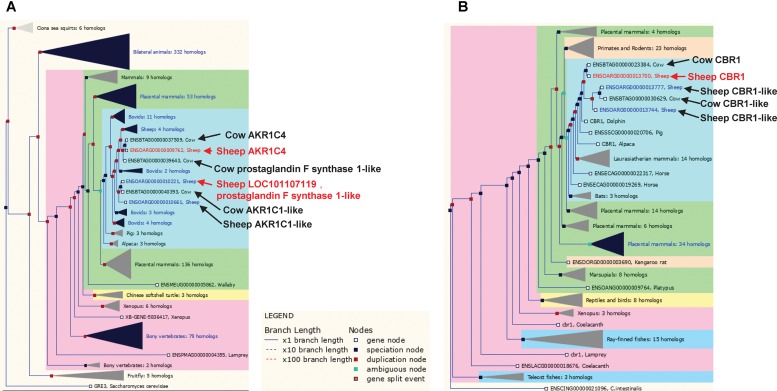
Trees for the aldo-keto reductase and short chain dehydrogenase/reductase gene families. **(A)** Aldo-keto reductase gene family, including sheep genes ENSOARG00000009762 and ENSOARG00000010221 (red text). **(B)** Short chain dehydrogenase/reductase gene family including the sheep gene ENSOARG00000013700 (red text). Other ruminant genes closely related to ENSOARG00000009762, ENSOARG00000010221, and ENSOARG00000013700 were also labeled in dark text.

The set of 14 metabolic genes also contained several poorly annotated enzyme encoding genes in families that have undergone significant gene duplication at the base of the ruminant evolution (e.g., sheep and cow), which likely have ruminant specific functions (**Figures 6A,B**). These genes include two members of the aldo-keto reductase family 1, ENSOARG00000009762 (annotated as *AKR1C4*) and ENSOARG00000010221 (annotated as LOC101107119, prostaglandin F synthase 1-like) and a carbonyl reductase family member ENSOARG00000013700 (annotated as carbonyl reductase 1, *CBR1*) (**Figure 6B** and **Table 2**). In the sheep transcriptome atlas of Clark et al. (2017), *AKR1C4* and *ENSOARG00000010221* also had very high tissue specificity in the rumen complex and all three genes were enriched in cellular metabolic functions (**Supplementary Table S4**). Since the rumen epithelium plays a key role in the uptake of SCFAs, which contribute to ketone-body synthesis (a component of the energy acquisition pathway in ruminants), the results of the current study suggest that *AKR1C* family members and *CBR1* may have important (but currently unknown) roles in the uptake of energy metabolites by the rumen wall.

### Gene Sets Associated With Rumen Concentrations of Short Chain Fatty Acids

Significant relationships between SCFA concentrations and CH_4_ production were observed in NZ experiment, but not in the AUS experiment (**Supplementary Table S2**). However, despite this difference a joint analysis was performed to identify genes with expression consistently correlated with individual SCFA concentrations in both the AUS and NZ datasets (**Figure 4**, **Supplementary Figure S5**). Overall, our approach identified the expression of 15 genes correlated with acetate concentration, 27 correlated with butyrate concentration and 31 correlated with propionate concentration in both AUS and NZ datasets. The mean expression of these genes and their regressions with SCFA concentration were shown in (**Supplementary Figure S5**). The cell cycle genes, representing rumen epithelial cell division, had the strongest and the most consistent positive relationships with SCFA concentrations. This is consistent with previous results where SCFAs in general stimulated rumen epithelial mitotic index (Sakata and Tamate, 1977; Sakata and Yajima, 1984). However, results in the current study showed only a small between-SCFA difference of relationships of individual SCFA concentrations with cell cycle gene expression (**Figure 4** and **Supplementary Figures S5A,B**). The observation that butyrate concentration was significantly correlated with cell cycle gene expression levels (**Supplementary Figure S5B**) is consistent with the proposed regulatory role of butyrate in rumen cell division (Sakata and Tamate, 1977, 1978).

Among the SCFAs, butyrate concentration had the strongest and most consistent correlations with the expression of genes from the metabolism gene set (**Figure 4** and **Supplementary Figure S5B**). These results are consistent with the conventional model that butyrate is the primary substrate for energy generation via ketone body metabolism in the rumen wall (Sejrsen et al., 2006). Many of the genes that had expression correlated with butyrate concentration were also correlated with CH_4_ traits (**Figure 4** and **Supplementary Figure S5B**). Interestingly, among the genes with the strongest and most consistent relationship with both SCFA concentrations and CH_4_ traits were *AKR1C4* and *LOC101107119* (**Figure 3**, red box), again highlighting their potential metabolic significance in ruminants.

The overall positive relationship between CH_4_ product and yield, and energy intake and the expression level of the metabolic gene set suggests non-competitive processes for substrates between microbial CH_4_ production and host energy acquisition via SCFA uptake in the rumen. Furthermore, stronger correlations of expression of metabolic genes with the produced CH_4_ than their correlations with SCFAs appears to support the hypothesis that the variations of the expression of these metabolic genes are driven by substrates produced by the rumen microbial fermentation, which at the same time generates CH_4_. In other words, the identified set of rumen metabolic genes may well be “effect” genes and thus unlikely to be involved in the “host control” processes of CH_4_ generation. The rumen epithelial metabolic process produces ketone bodies that contribute to the energy resources used by the host. The observed positive relationships between CH_4_ measurements and the expression of rumen metabolic gene sets also suggests a link between rumen CH_4_ emissions and the host energy intake, which cannot be ignored when selecting low CH_4_ emitting sheep.

### Rumen Muscle Genes Negatively Correlated With MRT and CH_4_ Yield

In sheep, several independent experiments have shown that shorter MRT leads to reduced CH_4_ yield (Goopy et al., 2014; Barnett et al., 2015). This is consistent with the observation from the present study of AUS animals (Bond et al., 2017; **Supplementary Figures S6A–C**) (MRT was not measured in NZ animals). In the AUS experiment, there were no significant correlations between MRT and the expression levels of any of the aforementioned major clusters of rumen genes, including the set of 14 metabolic genes that were associated with CH_4_ traits. Shorter MRT can be achieved by increased contractile forces of the rumen complex, driven by the rumen muscle components (Bueno and Ruckebusch, 1974; Okine et al., 1989; Okine and Mathison, 1991). Given the existence of a large number of genes of muscle origin consistently expressed in AUS and NZ rumen wall tissues (**Figures 1A,B**), the relationships between rumen muscle gene expression, MRT and CH_4_ yield were further explored by fully utilizing the available data from the two experiments. This was done by firstly identifying individual genes of rumen muscle that were negatively correlated with MRT in the AUS dataset (starting with 16,011 genes CPM value > 1 in all AUS samples) and then testing whether the expression of these AUS-MRT-associated genes were also negatively correlated with CH_4_ yield in both the AUS and the NZ datasets.

**FIGURE 7:**
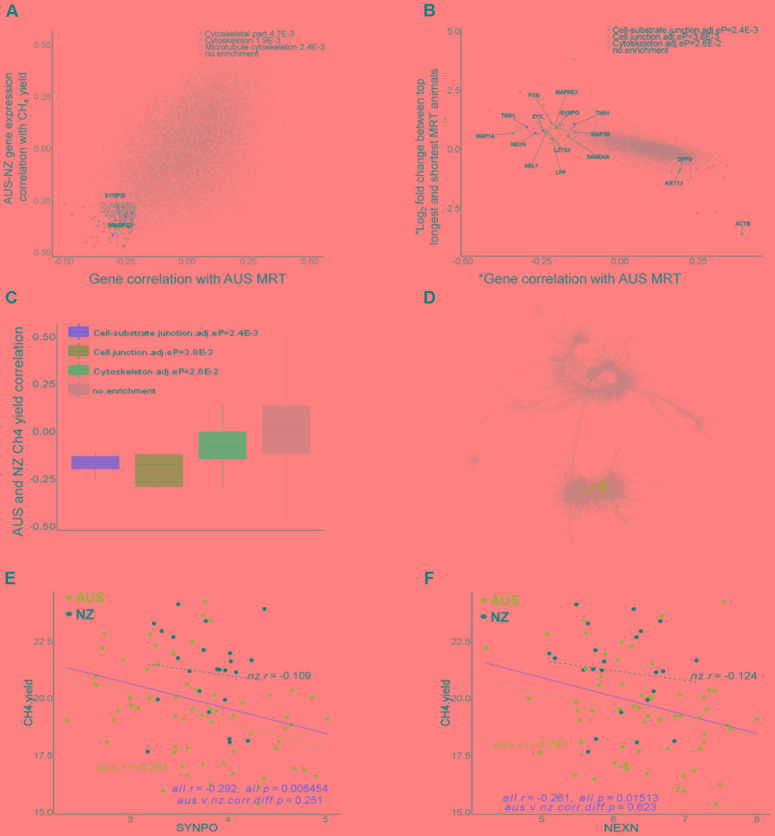
Genes associated with rumen feed mean retention time (MRT). **(A)** Transcriptome-wide relationships between the correlations of expression of individual genes with AUS MRT values and CH_4_ yield (g/day/dry matter intake, kg/d) in across the combined AUS and NZ datasets. The top 200 genes with negative correlations for both phenotypes were analyzed for DAVID enrichment and terms with adjusted enrichment *p*-values < 0.05 are indicated. **(B)** Transcriptome-wide relationships between the correlations of expression of individual genes with AUS MRT values and differential gene expression between the top 10 shortest and longest MRT animals in the AUS dataset. ^∗^ indicates the transcriptome data was normalized for the mean expression of muscle genes present in both the AUS and NZ gene network to remove the effect of the composition of the full thickness rumen wall samples. The genes indicated were analyzed for DAVID enrichment and terms with adjusted enrichment *p*-values < 0.05 are shown. The list of genes with their *p*-values and FDR for differential expression between long and short MRT animals can be found in **Supplementary Table S6**. **(C)** Expression of genes in enriched pathways in **(B)** were plotted for their correlations with CH_4_ yield across both the AUS and NZ datasets. **(D)** Location of genes showing significant differential expression between long and short MRT animals and with negative correlations (within top 200) with MRT in the AUS-NZ combined network. For genes with enrichments their colors correspond to dot colors in panel **(B)**. **(E,F)** Scatter plots of gene expression correlations with CH_4_ yield in the animals in the AUS and NZ datasets for example genes from the set of genes identified in the analysis above.

In the set of the top 200 genes whose expression levels were negatively correlated with both MRT in the AUS dataset (*r* ranged from -0.5 to -0.21) and CH_4_ yield in the AUS and NZ datasets (*r* ranged from -0.47 to -0.26), 21 showed significant enrichments of biological pathways in DAVID (Huang da et al., 2009) with Benjamini-Hochberg adjusted *p*-values < 0.05 (**Figure 7A**), including the cytoskeleton function (**Figure 7A**). Later we also showed that 46 out of these 200 genes had differential expression (DE) between long and short MRT animals with FDR < 0.1 (**Supplementary Table S6**). The cytoskeleton is a key component of the structure of muscle, which has a specific cytoskeleton associated with it and many of the genes are related to cytoskeletal proteins of the myofibrils (Henderson et al., 2017). Muscle does not have such a high cell turnover rate as the epithelium cluster. This is consistent with differences in MRT being more likely to be due to differences in the muscle layers than in the epithelial layers of the rumen wall. These results indicated the existence of muscle signals in this dataset correlated with MRT and this was the motivation for the following differential gene expression analyses between long and short MRT animals. The rumens in the animals in this analysis varied about 3.5-fold in the actual thickness of the muscle layer of the rumen wall (**Supplementary Figure S7**). However, there was no significant correlation between the thickness of the rumen wall and MRT in the AUS dataset (**Supplementary Figure S7**).

Next, we investigated whether a difference in the composition of the muscle in the rumen wall, as represented by gene expression, may be related to MRT. Since variation in the proportion of muscle in the full thickness rumen wall samples is the major driver of variation in the observed muscle gene expression, further normalization of the data was required to enable to address this question. The gene expression data was normalized using the average expression of a large set of genes included in the muscle cluster in all three global gene expression networks to minimize the variation due to difference in proportions of muscle in the samples and to maximize any signals of difference in composition per unit volume of muscle in the rumen wall. Firstly, we compared rumen wall gene expression of the top 10 shortest and top 10 longest MRT animals (**Supplementary Figure S6D**) in the muscle expression normalized datasets. This led to the identification of 236 genes with DE at the FDR < 0.1 level when fitting the mean muscle gene expression as a covariate (**Supplementary Table S6**). Of these 236, 87 of them also had DE at the FDR < 0.1 level when fitting the rumen muscle depth as a covariate (the 8th column of **Supplementary Table S6**). In total, there were 88 genes with DE at FDR < 0.1 level when fitting the rumen muscle depth as a covariate. Thus, the results from the DE fitting the mean muscle gene expression as a covariate included most of the results from the DE fitting the mean depth as a covariate. As muscle depth was not available in NZ dataset but the mean expression of the rumen muscle genes was based on both AUS and NZ datasets, we preferred to use the results fitting the mean muscle gene expression as a covariate in the following sections.

To refine the gene list, we prioritized 41 genes with significant DE (FDR < 0.1, fitting the mean muscle gene expression as a covariate) which also were within the top 200 of genes with expression negatively correlated with MRT (**Supplementary Table S7**). Such integrative analysis allowed us to identify genes whose changes in expression were associated with variation in MRT that were significantly enriched in cell junction and cytoskeleton functions (**Figure 7B**). All of these genes were located in the established muscle cluster (**Figure 7C**) and had negative correlations with CH_4_ yield in both the AUS and NZ datasets (**Figures 7D–F**). The list of 41 genes identified with their significant DE information and their correlation with MRT and CH_4_ yield is displayed in **Supplementary Table S7**. Several of the identified genes with biological relevance are candidates for a role in host control of MRT and hence CH_4_ yield (**Figures 7D–F**). For example, *SYNPO* (synaptopodin) encodes an actin-associated protein which plays a role in actin-based cell shape and motility (Kremerskothen et al., 2005). NEXN (nexilin) plays an important role in mammal cardiac development (Yang et al., 2014) and F-actin binding. *SYNPO* and *NEXN* are all involved in the cell junction pathway (**Figure 7B**), an important feature of smooth muscle regulating contraction (Eddinger et al., 2005). *KCNMA1* (Potassium Calcium-Activated Channel Subfamily M Alpha 1) encodes an integral membrane bound protein forming voltage and calcium-sensitive potassium channels which are fundamental to the control of smooth muscle tone and neuronal excitability (refseq). Although *KCNMA1* was not included in the final list (**Supplementary Table S7**), it showed significant DE between long and short MRT animals (FDR < 0.05, **Supplementary Table S6**). It is a known regulator of smooth muscle contraction (Ghatta et al., 2006). The increased expression in the rumen of these genes and the pathways/structures in which they are involved may increase gastrointestinal muscle contraction, which is predicted to lead to more rapid feed passage (i.e., reduced MRT) and consequently contribute to reduced CH_4_ yield. Thus, our results reveal some attractive candidates to select for increasing smooth muscle contraction rates in the rumen wall, which may decrease MRT and consequently mitigate CH_4_.

Our study has potential limitations, including that the analyses were largely based on correlations between gene expression and phenotype which do not necessarily imply causation. In addition, the activity of proteins, especially regulatory proteins, may not necessarily be reflected in the level of expression of the encoding genes in the tissue. Some of the identified host gene-to-phenotype correlations may also be contributed by rumen microbial populations, which requires future integrative studies with the host factors and CH_4_ phenotype. Also, HTseq (Anders et al., 2014) focusing on uniquely mapped reads was used to generate our gene count data, which might lead to false negative errors due to filtering out potentially informative genes with multi-mapped reads (Robert and Watson, 2015). While uniquely mapped reads are still widely used in understanding the relationships between gene expression and complex traits (Consortium, 2017; Bouwman et al., 2018), utilizing multi-mapped reads as suggested by (Robert and Watson, 2015) in the future research may lead to new discovery of gene candidates associated with CH_4_ phenotypes. We have analyzed the CH_4_ phenotypes one at a time and future analyses modeling these variables in a single model using multivariate approaches (Lê Cao et al., 2009; Rohart et al., 2017) may provide additional efficiency and insights into the analyses of complex CH_4_ phenotypes.

## Conclusion

We combined transcriptomic datasets from two completely independent experiments conducted in AUS and NZ, where feeding regime, animals, and CH_4_ phenotype are significantly different. We compared relationships between the expression of rumen wall gene clusters and CH_4_ phenotype across experiments and the results significantly enhanced our understanding of the host rumen mechanisms associated with enteric CH_4_ emissions. The side-by-side between-experiment transcriptomic comparisons validated core rumen gene sets representing major epithelial and the muscular functions. While cell cycle genes are always positively correlated with SCFA concentrations, a group of metabolic genes are always positively correlated with CH_4_ and butyrate concentrations, included key ketone body metabolism members. This set of metabolic genes also contained poorly annotated sheep genes members of the AKR1C family with probable ruminant origin, highlighting their probable significance in rumen metabolism. The strong relationship between metabolic genes and CH_4_ emission suggests a tight relationship between enteric CH_4_ and the host energy metabolism. Sheep with shorter MRT (negatively related with lower CH_4_ yield) had higher gene expression levels of genes encoding proteins involved in cell junctions in the muscle component of the rumen wall, which are an important feature of smooth muscle contraction. Increased rumen contraction likely reduces MRT and consequently CH_4_. These genes might therefore be targets to select hosts for mitigating CH_4_ and possibly maintaining digestion efficiency. The expression properties of these targets may allow for genetic selection. Future research of the impact of these rumen gene clusters on the activities of methanogens may further our understanding of the detailed mechanisms of host control of CH_4_ emissions.

## Materials and Methods

### Animals, Experimental Design, and Measurements

The Australian animal experiment was conducted at the University of New England, Armidale, Australia, in accordance with the University of New England Animal Ethics Committee (AEC no14-041) and the animal experiment in New Zealand was conducted at AgResearch Grasslands Research Center, Palmerston North, New Zealand with approval in accordance of AgResearch Code of Ethical Conduct. Full details of the experimental design have been reported by (Bond et al., 2017) for the Australian experiment and by Xiang et al. (2016a) for the experiment in New Zealand. Summary of experimental conditions is displayed in **Table 1**. The Australian experiment was performed with 64 ewes (∼3 years of age) of four Merino sires in an incomplete randomized block design with 16 sheep per block (i.e., test period of 4 weeks). All sheep were fed lucerne:oat chaff (50:50) at 1.5 × maintenance metabolizable energy (ME, MJ/kg DM) requirements (Bond et al., 2017). ME for the AUS sheep was 9.9 MJ/kg DM (Bond et al., 2017) and this was used to calculate the DE with the equation: DE Mcal/kg = ME Mcal/kg/0.82, based on UC Davis energy conversion calculator^1^. The experiment in New Zealand was performed with 24 composite breed ewe lambs (∼9 months of age) of low and high CH_4_ yield selection line sires in one period. Animals were fed good or poor quality cut ryegrass based pasture at 1.0 or 1.5 × maintenance ME (MJ/kg DM) requirements (Xiang et al., 2016a). The DE of NZ sheep was calculated using the equation: DE Mcal/kg = Gross energy (MJ/kg DM) × 0.001 × *in vitro* DM digestibility (g/kg DM). For NZ sheep with good quality feed, the gross energy and *in vitro* DM digestibility were 17.8 MJ/kg DM and 686 g/kg DM, respectively. For NZ sheep with poor quality feed, the gross energy and *in vitro* DM digestibility were 17.6 MJ/kg DM and 659 g/kg DM, respectively.

In both experiments, after adjustment to the dietary conditions, animals were measured in the respiratory chambers to measure CH_4_ production and dry matter intake for 48 h and a single rumen sample was collected from SCFA measurement. In addition, in the Australian experiment, measurements of rumen feed MRT were estimated using inert markers over a 7-day period in combination with excretion curve fitting to compartment model, and CT scanning to determine rumen volume were performed (Bond et al., 2017). The animals in both experiments were sacrificed at the end of the experimental periods. During the course of the experiment in AUS, two sheep, both in block 3, were lost from the experiment (Bond et al., 2017).

### RNA Preparation and Sequencing

After sacrifice of the animals, the whole ventral rumen wall of each sheep was sampled and placed in RNALater^®^-ICE Frozen Tissue Transition Solution (Ambion^®^) and stored at -20°C. The RNA extraction and sequencing protocols followed previous study (Xiang et al., 2016a). Briefly, rumen wall samples were later transported to CSIRO’s FD McMaster Laboratory at Chiswick, Armidale, Australia for extraction. RNA of 62 animals was extracted from 200 mg ventral rumen tissue using the Qiagen RNeasy^®^ Midi Kit with on-column DNase digestion. The average RNA concentration was measured using a Quant-iT^TM^ RiboGreen^®^ RNA reagent and kit (Invitrogen^TM^) on a fluorescent plate reader. Samples of ∼4 μg of total RNA were rRNA depleted using the Ribo-Zero^TM^ Magnetic Gold Kit (Human/Mouse/Rat) (Epicenter^®^) and purified using a Qiagen RNeasy^®^ MinElute Cleanup Kit. A total of 12 μl rRNA depleted RNA was sent to the Ramaciotti Genomics Center, The University of New South Wales, Australia. RNA Quality was checked and stranded libraries were prepared using SureSelect^TM^ stranded RNA sample preparation kit (Agilent Technologies) at the sequencing center. RNA sequencing was performed as 100 base paired-end reads in two lanes of Illumina HiSeq2000 instrument housed at the Ramaciotti Genomics Center. Total reads were ranging from 15 to 20 million per sample. RNA sequencing results were checked for quality using FastQC at the sequencing center. A total of 16,011 genes with CPM value > 1 = in all samples of the AUS data were retained.

### Sequence Data Analysis

The AUS ventral rumen wall RNA sequencing raw reads files (fastq) were retrieved from the sequencing center. The NZ ventral rumen transcriptomic data was generated previously (Xiang et al., 2016a) (accession PRJNA313132). Raw reads were aligned to Ensembl sheep gene models v3.2 (Ensembl Genes release 78) with additional models for genes at the epidermal differentiation complex locus (Jiang et al., 2014) using Tophat v2.1.0 (Trapnell et al., 2009) and bowtie2 v2.1.0 (Langmead and Salzberg, 2012) with default options. BAM files were calculated for counts using HTSeq v0.6.1 in the Python environment (Anders et al., 2014).

### Construction of Gene Expression Networks

A hypothesis-free global gene expression network of the Australian data (AUS) was constructed to understand general gene expression patterns across the studied animals. Transcripts with at least 3-CPM in all 62 rumen samples were normalized by voom (Law et al., 2014) and transformed expression values were analyzed for co-expression using PCIT (Reverter and Chan, 2008) in R v3.1.4. To reduce the complexity of the network the PCIT output was filtered for pairs of genes with a correlation coefficient > 0.8 and visualized in Cytoscape v3.1.2 (Shannon et al., 2003). The identification of gene sub-clusters followed previous procedures (Xiang et al., 2016a) using the Glay community clustering plugin in Cytoscape (Shannon et al., 2003). To demonstrate the consistency between NZ and AUS ventral rumen transcriptome data, AUS global network was mapped to the NZ global gene expression network based on appearances of genes in respect networks. An AUS-NZ combined global gene expression network was also generated by combining voom transformed AUS and NZ transcriptomic data and normalizing together using the quantile method, implemented in the Affy package (Bolstad et al., 2003). After voom transformation, the RNA sequence count data has properties similar to microarray data (Law et al., 2014). This allowed the application of quantile normalization, a method that has been widely used to remove sources of variation between arrays of non-biological origin from microarray data, to our RNA sequence data. The jointly normalized AUS-NZ data were also analyzed using PCIT and visualized in Cytoscape by the same procedures described above. The hypergeometric distribution (hP) (Andrews et al., 1999) was used to determine significance of overlaps of genes in the different datasets.

### Correlation Analysis

For gene sets present in both the AUS and NZ gene expression networks (**Figure 1A**), the mean expression of the genes in the cluster were calculated for each animal in the AUS and NZ datasets based on AUS-NZ jointly normalized expression data. Then mean expression of a gene set was analyzed for correlation with respect AUS and NZ phenotype. All phenotype data were not normalized as these phenotypes, such as CH_4_ production (g/day) and yield (g/d/DMI) are well used parameters in different sheep studies (Goopy et al., 2014, 2016; Shi et al., 2014). The significance of between-experiment differences of correlations of gene expression with phenotype was determined by Fisher’s Z method (review and integrated in (Lui et al., 2015): for AUS correlation *r_aus_*(*f,i*) and NZ correlation $r_{nz}(f,i):z_{aus}=\frac{1}{2}$ ln $\left(\frac{1+r_{aus}}{1-r_{aus}}\right)$ and $z_{nz}=\frac{1}{2}$ ln $\left(\frac{1+r_{nz}}{1-r_{nz}}\right)$, respectively. This allowed for normal distribution and calculation of the standard error based Z-differences between correlation coefficients in AUS and NZ animals: $z_{aus-nz}=\frac{\left|z_{aus}-z_{nz}\right|}{\sqrt{\frac{1}{n_{aus}-3}+\frac{1}{n_{nz}-3}}}.$ *n_aus_* and *n_nz_* represented sample size in AUS and NZ groups, respectively. *z_aus-nz_* then led to significant tests of difference of correlation coefficients which were estimated by pnorm function in R v3.14.

While there were a number of significant differences of design and feeding regime between AUS and NZ experiments, the overall rumen wall gene expression patterns in two experiments showed consistency. To identify gene sets showing consistent correlations with CH_4_ production (g/d) and yield (g/kg DMI) in both AUS and NZ dataset, a stepwise approach based on both gene-phenotype and gene-gene relationships was used. This approach considered the differences between experiments as the negative control for unreproducible gene-to-phenotype and gene-gene correlations in two independent studies. All calculations were performed in R v3.14 if not specified. For each dataset, Pearson correlation coefficient between each voom transformed gene expression value and CH_4_ production and yield was calculated. Gene-phenotype correlations with absolute coefficient within upper 1/3 quartile were chosen to perform PCIT analysis to construct gene network using Cytoscape based on pairwise gene-gene correlations > 0.8. The concept behind this approach is that if a gene set is important for a phenotype, member genes within this gene set should be involved in similar pathways thus should be well correlated with each other. AUS and NZ dataset were analyzed separately for this approach and the results (AUS and NZ networks containing genes well correlated with each other and with network individual gene member reasonably correlated with the phenotype) were matched based on: (1) appearance of genes (network nodes) in both AUS and NZ network, (2) showing the same correlation sign with the trait in both AUS and NZ data, and (3) the absolute value of correlation coefficient should be no less than 0.15 in both datasets. The cutoff of absolute correlation coefficient > 0.15 was an empirical choice to filter out noisy correlations. Our previous studies have shown that the gene selection based on ranking of correlations between gene expression and phenotype is more informative than the selection based on p-value of the correlation (Guo et al., 2014, 2015). As SCFAs were also important parameters associated with CH_4_ traits, the same procedures described were also applied to analyze individual SCFA concentrations in both AUS and NZ dataset.

To explore the expression of genes potentially correlated with MRT, correlations of the whole transcriptome expression of AUS and NZ CH_4_ yield and AUS MRT was plotted (**Figure 7A**). Based on the ranking of gene-phenotype correlations the top 200 genes showing negative correlations with both CH_4_ yield and AUS MRT were selected for enrichment analysis with DAVID (Huang da et al., 2009). Only enriched terms with Benjamini-Hochberg adjusted *p*-values < 0.05 accounting for multi-testing as implemented in DAVID (Huang da et al., 2009) were considered. To further identify expression of genes associated with MRT, which was associated with CH_4_ yield in the study of (Bond et al., 2017) and in previous experiments (Pinares-Patiño et al., 2003; Goopy et al., 2014), a subset of data from 20 animals with the highest and the lowest MRT values were selected (**Supplementary Figure S6D**). Gene DE was analyzed in these 20 animals using edgeR (Robinson et al., 2010) with the following linear model: *y* = *block* + *muscle.gene* + *MRT.status* + *e* (1). The *block* variables had four levels (block 1, 2, 3, 4); the *muscle.gene* is a continuous variable, which is the mean gene expression value of members of the rumen major structural muscle gene cluster that appeared both in AUS and NZ global network described above; the *MRT.status* variable had two levels (short and long). Variation in MRT is associated with changes in rumen muscle physical contractile activities rather than changes in the rumen muscle amounts (Okine and Mathison, 1991), which are more likely to be captured by gene expression assay. To capture changes in the rumen muscle physical activity between short and long MRT animals at the gene expression level, the mean expression of the cluster of rumen muscle genes, which appeared in both AUS and NZ networks, were fitted in the linear model. Fitting the average muscle gene expression in the model as a covariate allowed comparisons between long and short MRT animals based on the adjustment where these animals had the same mass of rumen muscle. This enables us to identify variations in expression of genes representing “regulators” that can lead to changes in rumen muscle physical activities. To further filter the DE results, we normalized the whole transcriptome to the mean gene expression value of the AUS-NZ included major structural muscle gene cluster, which has been used in gene expression analysis before (Jaeger and Spang, 2006). Normalized transcriptome data of muscle gene expression were used to calculate individual correlations with MRT. We also performed DE analysis, fitting muscle depth as a covariate: *y* = *block* + *muscle.depth* + *MRT.status* + *e* (2), where *muscle.depth* is the rumen muscle depth measured for studied animals. The DE results from model (2) were compared with results of model (1) and there was a significant overlap of results. The final list of genes associated with MRT was defined as within FDR of MRT DE < 0.1 (from model (1) and after muscle gene cluster normalization within top 200 negative correlations with MRT (**Figure 7B**). The location of these genes in the combined AUS-NZ network were mapped (**Figure 7D**). The correlation of the original expression of these genes (not normalized by muscle gene expression, **Supplementary Table S1**) with CH_4_ yield were also calculated (**Figures 7C,E,F**).

## Availability of Data and Materials

The AUS RNA sequencing dataset is deposited in the NCBI GEO database with the accession number GSE81847. The NZ RNA sequencing dataset is deposited at NCBI Sequence Read Archive with the accession number PRJNA313132. The R codes used in the paper are available at https://github.com/rxiangr/sheep-ch4.

## Ethics Statement

The animal studies were approved and carried out in accordance with the Animal Ethics Committees of NSWDPI [AUS experiment (Bond et al., 2017)] and AgResearch Grasslands [NZ experiment (Xiang et al., 2016a)].

## Author Contributions

JCM, CP-P, AJ, and SR designed the NZ animal experiment. VO, JB, and PV designed the AUS experiment. JCM, SR, CP-P, and AJ led the rumen sampling of the NZ animals. JB, DT, MC, AD, and KA participated in the rumen sampling of AUS animals. JCM and JM prepared the RNA from the all rumen wall samples. RX and BD designed the analysis. RX, BD, JB, and VO performed the analyses. RX and BD wrote the manuscript. RX, BD, JB, VO, AJ, and JCM revised the manuscript. All the authors read and approved the final manuscript.

## Conflict of Interest Statement

The authors declare that the research was conducted in the absence of any commercial or financial relationships that could be construed as a potential conflict of interest.

## Footnotes

1 http://animalsciencey.ucdavis.edu/java/LivestockSystemMgt/Conversion/energy.htm

## Supplementary Material

FIGURE S1 The relationship between CH4 production and yield plotted using AUS and NZ data together.

FIGURE S2 PCA plot of transcriptome data of AUS and NZ with data generated by Roslin institute.

FIGURE S3 NZ rumen transcriptomic network.

FIGURE S4 Epithelial and cell cycle gene expressions in different blocks of AUS animals.

FIGURE S5 The regression of concentrations of principal short chain fatty acids on the expression of the metabolic gene set in AUS and NZ experiments.

FIGURE S6 Relationships between CH4 production, CH4 yield, dry matter intake (DMI) and mean retention time (MRT) in the AUS experiment.

FIGURE S7 Relationships between rumen muscle parameters and CH4 yield and MRT.

TABLE S1 Difference of CH_4_ parameters between AUS and NZ experiments.

TABLE S2 Correlation coefficients (*r*) between CH_4_ and rumen parameters within AUS and NZ experiments.

TABLE S3 Overlapped genes between AUS and NZ network.

TABLE S4 The annotation data from the latest sheep transcriptome atlas (Clark et al., 2017) for the genes involved in ketone body metabolic pathways and genes correlated with methane phenotype.

TABLE S5 Gene expression correlation coefficients (*r*) with CH_4_ and rumen phenotype in AUS and NZ experiments.

TABLE S6 Genes showed differential gene expression with FDR < 0.1 between long and short MRT animals in the AUS experiment.

TABLE S7 Prioritized 41 rumen muscle gene candidates associated with MRT in the AUS experiment.

## Abbreviations

AUS: Australian
MRT: Mean Retention Time
NZ: New Zealand
