# Supplementary material for: Across-Experiment Transcriptomics of Sheep Rumen Identifies Expression of Lipid/Oxo-Acid Metabolism and Muscle Cell Junction Genes Associated With Variation in Methane-Related Phenotypes
Source: Front Genet. 2018 Aug 20;9:330. doi: 10.3389/fgene.2018.00330 (PMC6109778; doi:10.3389/fgene.2018.00330)
Supplement: FIGURE S1 — The relationship between CH4 production and yield plotted using AUS and NZ data together. [file Image_1.PDF]

Figure S1

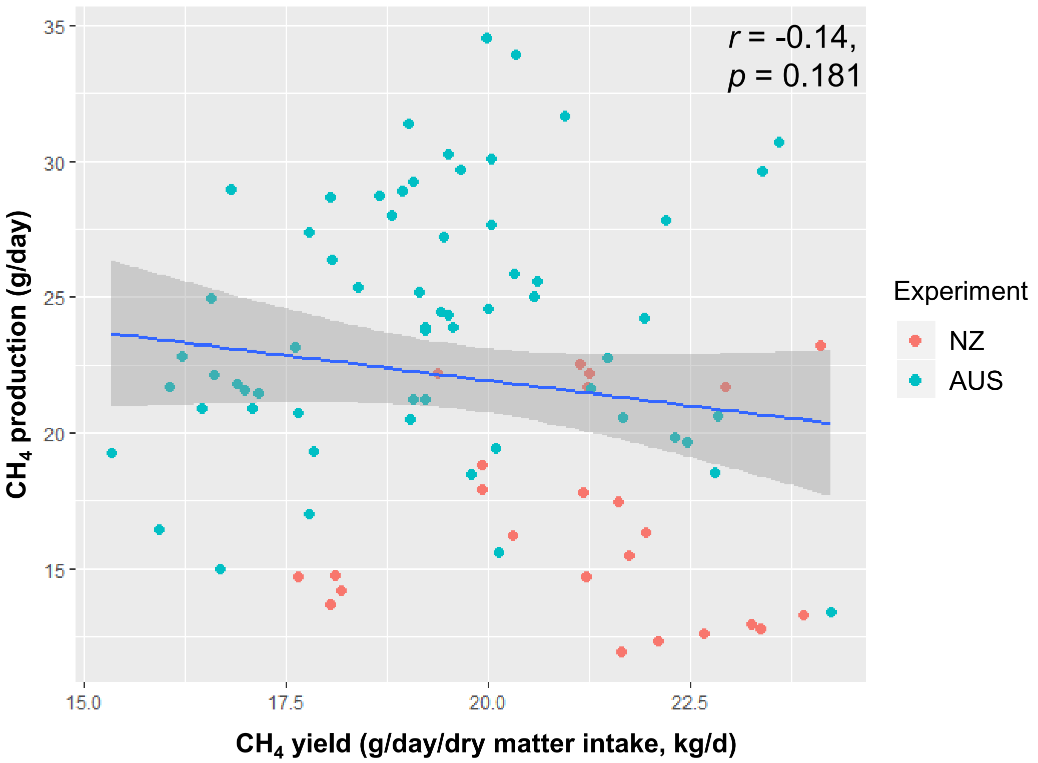

Figure S1. The relationship between CH_4 production and yield plotted using Australian (AUS) and New Zealand (NZ) data together. Correlation coefficients (*r*) and significance (*p*)of correlation were indicated.
