# Supplementary material for: Across-Experiment Transcriptomics of Sheep Rumen Identifies Expression of Lipid/Oxo-Acid Metabolism and Muscle Cell Junction Genes Associated With Variation in Methane-Related Phenotypes
Source: Front Genet. 2018 Aug 20;9:330. doi: 10.3389/fgene.2018.00330 (PMC6109778; doi:10.3389/fgene.2018.00330)
Supplement: FIGURE S2 — PCA plot of transcriptome data of AUS and NZ with data generated by Roslin institute. [file Image_2.pdf]

Figure S2

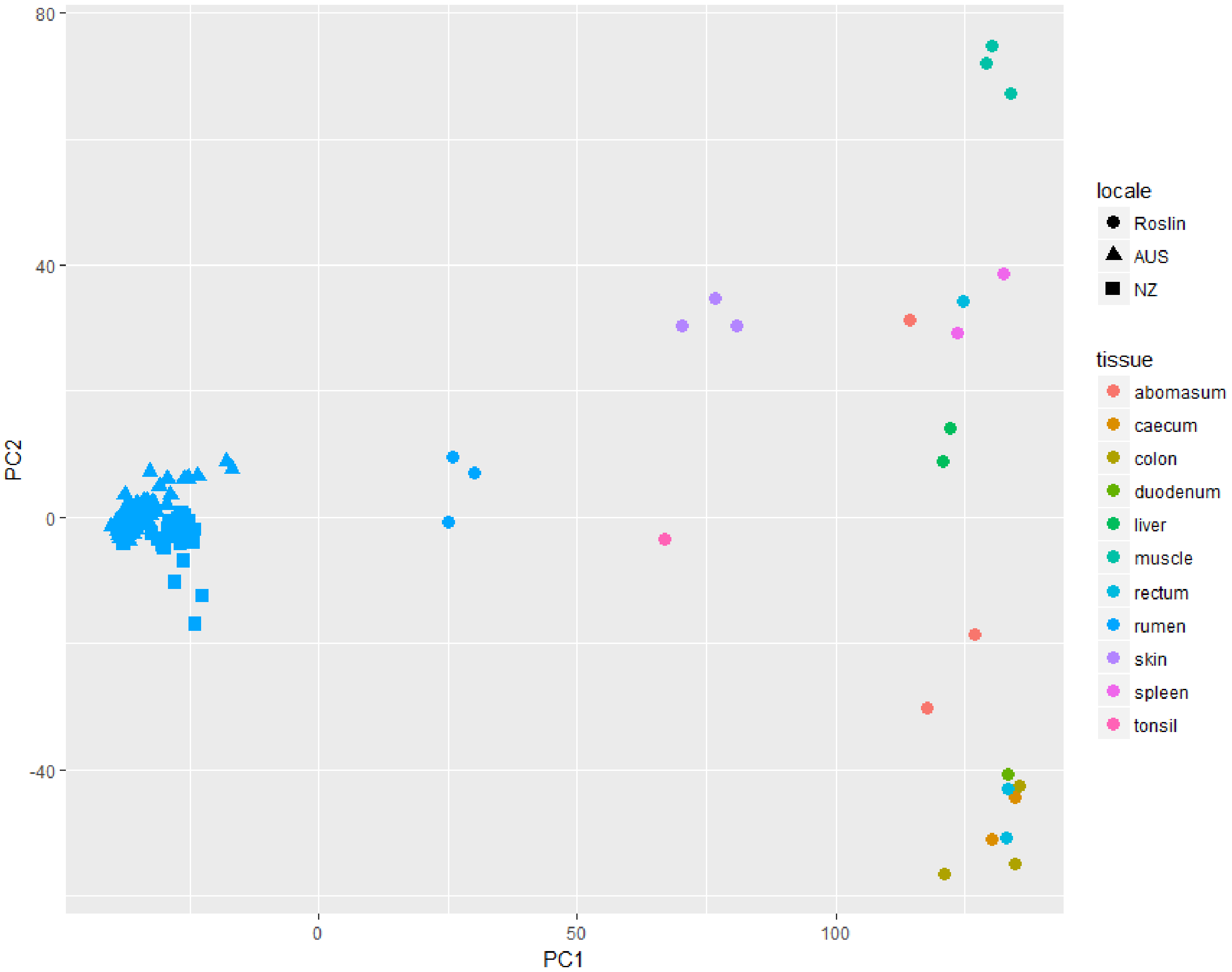

Figure S2: **PCA plot of transcriptome data of AUS and NZ with data generated by Roslin institute in studies of [1] and [2].** PCA data were calculated using DESeq2 [3] using default settings.

[1] Jiang, Y., Xie, M., Chen, W., Talbot, R., Maddox, J.F., Faraut, T., et al. (2014). The sheep genome illuminates biology of the rumen and lipid metabolism. *Science* 344(6188)**,** 1168-1173.

[2] Xiang, R., Oddy, V.H., Archibald, A.L., Vercoe, P.E., and Dalrymple, B.P. (2016). Epithelial, metabolic and innate immunity transcriptomic signatures differentiating the rumen from other sheep and mammalian gastrointestinal tract tissues. *PeerJ* 4**,** e1762.

[3] Love, M.I., Huber, W., and Anders, S. (2014). Moderated estimation of fold change and dispersion for RNA-seq data with DESeq2. *Genome biology* 15(12)**,** 550.
