# Supplementary material for: Across-Experiment Transcriptomics of Sheep Rumen Identifies Expression of Lipid/Oxo-Acid Metabolism and Muscle Cell Junction Genes Associated With Variation in Methane-Related Phenotypes
Source: Front Genet. 2018 Aug 20;9:330. doi: 10.3389/fgene.2018.00330 (PMC6109778; doi:10.3389/fgene.2018.00330)
Supplement: FIGURE S3 — NZ rumen transcriptomic network. [file Image_3.pdf]

Figure S3

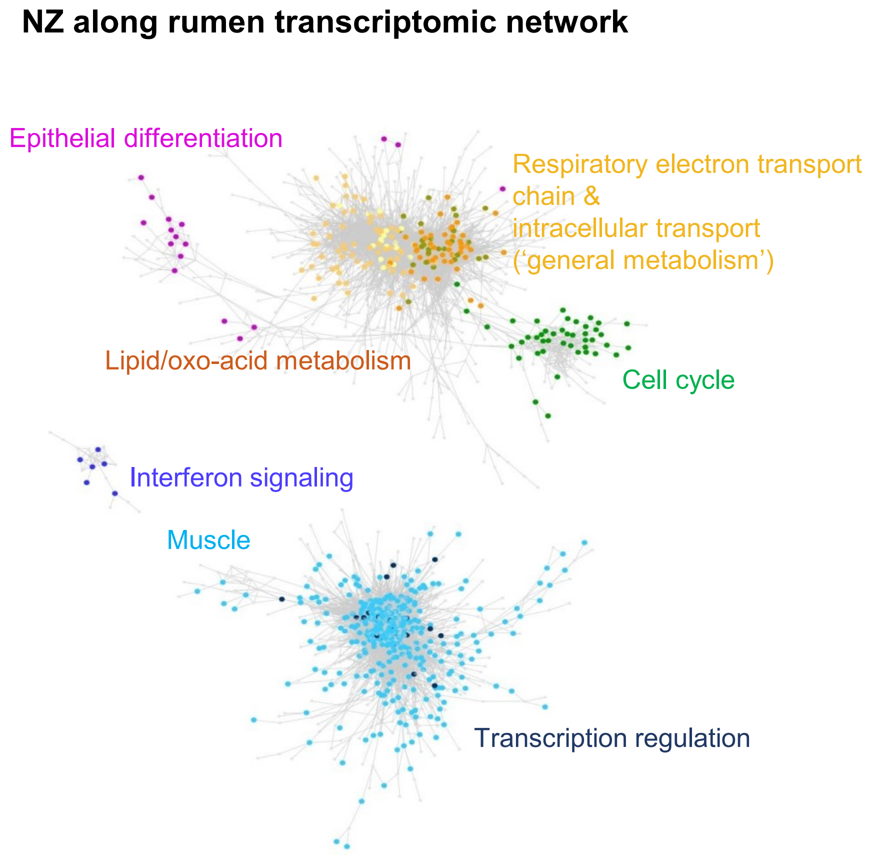

Figure S3: Gene network reproduced using data from the New Zealand CH_4 experiment [1].

[1] Xiang, R. *et al.* Gene network analysis identifies rumen epithelial cell proliferation, differentiation and metabolic pathways perturbed by diet and correlated with methane production. *Sci Rep* **6** (2016).
