# Supplementary material for: Across-Experiment Transcriptomics of Sheep Rumen Identifies Expression of Lipid/Oxo-Acid Metabolism and Muscle Cell Junction Genes Associated With Variation in Methane-Related Phenotypes
Source: Front Genet. 2018 Aug 20;9:330. doi: 10.3389/fgene.2018.00330 (PMC6109778; doi:10.3389/fgene.2018.00330)
Supplement: FIGURE S4 — Epithelial and cell cycle gene expressions in different blocks of AUS animals. [file Image_4.pdf]

Figure S4

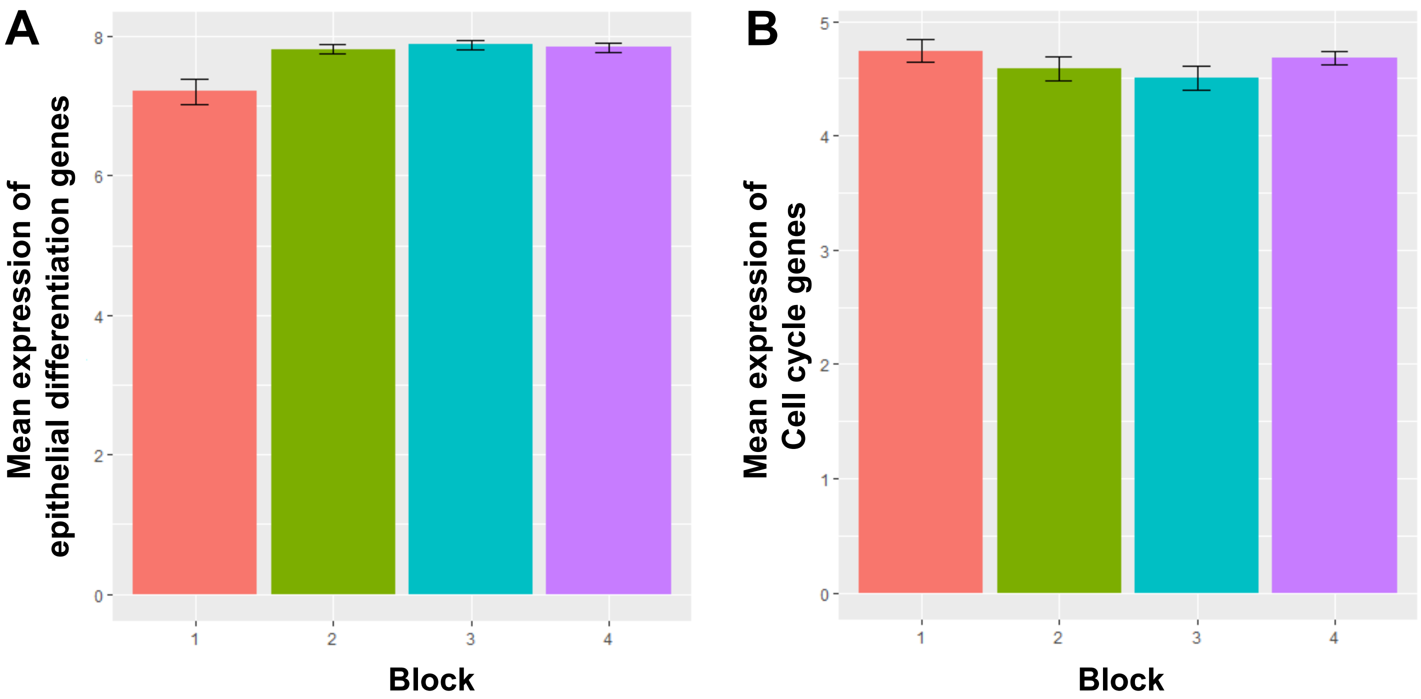

Figure S4: **The mean and standard error of expression of genes from the epithelial differentiation and cell cycle clusters.** The average expression of epithelial gene sets of the animals from block 1 was lower than the animals from other groups (**A**). However, such effects are not significant on cell cycle genes (**B**).
