# Supplementary material for: Across-Experiment Transcriptomics of Sheep Rumen Identifies Expression of Lipid/Oxo-Acid Metabolism and Muscle Cell Junction Genes Associated With Variation in Methane-Related Phenotypes
Source: Front Genet. 2018 Aug 20;9:330. doi: 10.3389/fgene.2018.00330 (PMC6109778; doi:10.3389/fgene.2018.00330)
Supplement: FIGURE S5 — The regression of concentrations of principal short chain fatty acids on the expression of the metabolic gene set in AUS and NZ experiments. [file Image_5.pdf]

Figure S5

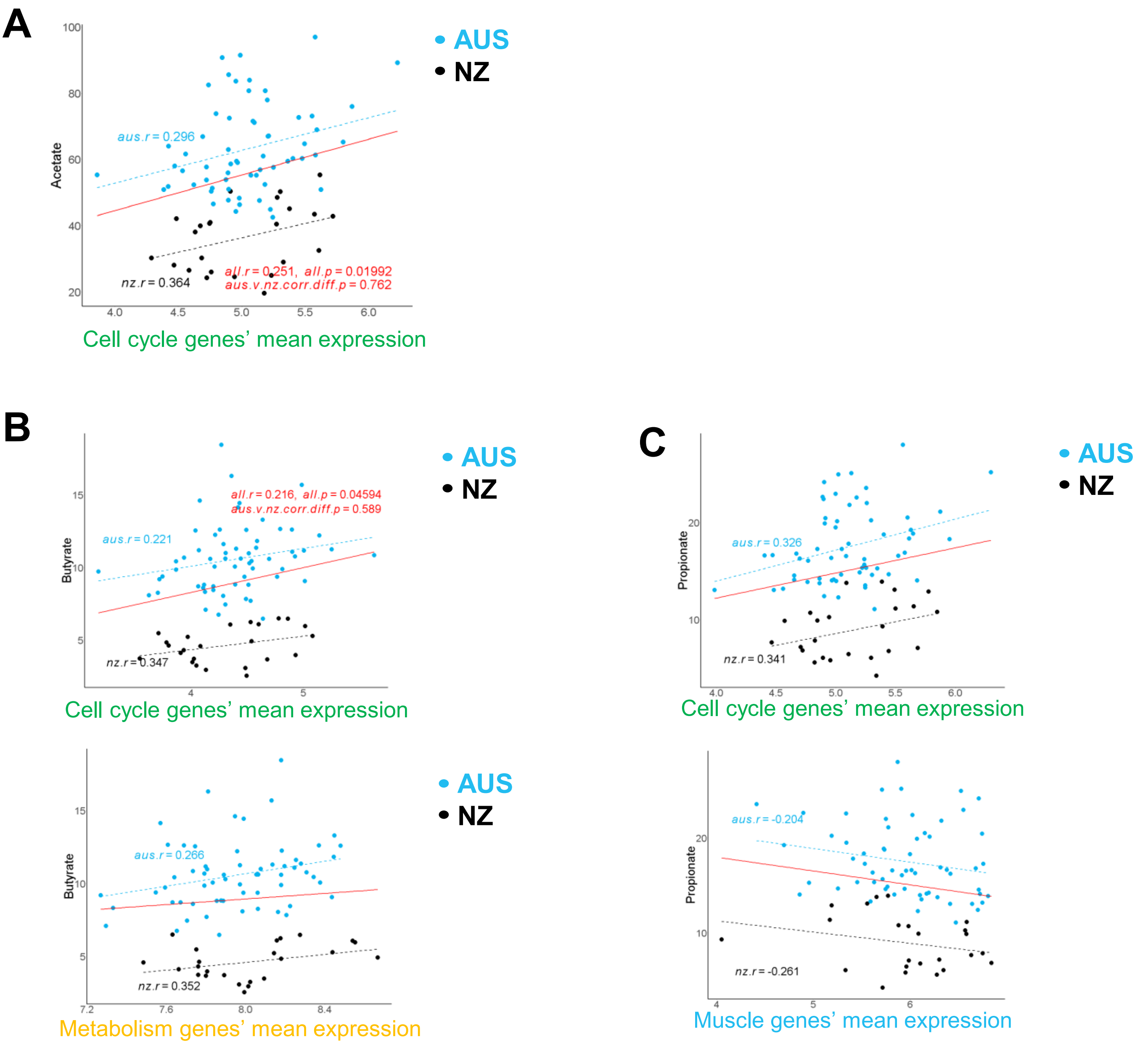

Figure S5. The consistent regressions of concentrations of principal short chain fatty acids on the expression of the metabolic gene set in Australian (AUS) and New Zealand (NZ) experiments. Regressions were shown for acetate (**A**), butyrate (**B**) and propionate (**C**). Significance of between-experiment difference of correlations for mean gene cluster expressions were indicated. Units for phenotypes values are: CH4.yield the CH_4 yield unit is (g/day/dry matter intake), CH4.prod (g/d), DMI (kg/d) and concentration of acetate, butyrate and propionate (mM).
