# Supplementary material for: Across-Experiment Transcriptomics of Sheep Rumen Identifies Expression of Lipid/Oxo-Acid Metabolism and Muscle Cell Junction Genes Associated With Variation in Methane-Related Phenotypes
Source: Front Genet. 2018 Aug 20;9:330. doi: 10.3389/fgene.2018.00330 (PMC6109778; doi:10.3389/fgene.2018.00330)
Supplement: FIGURE S6 — Relationships between CH4 production, CH4 yield, dry matter intake (DMI) and mean retention time (MRT) in the AUS experiment. [file Image_6.pdf]

Figure S6

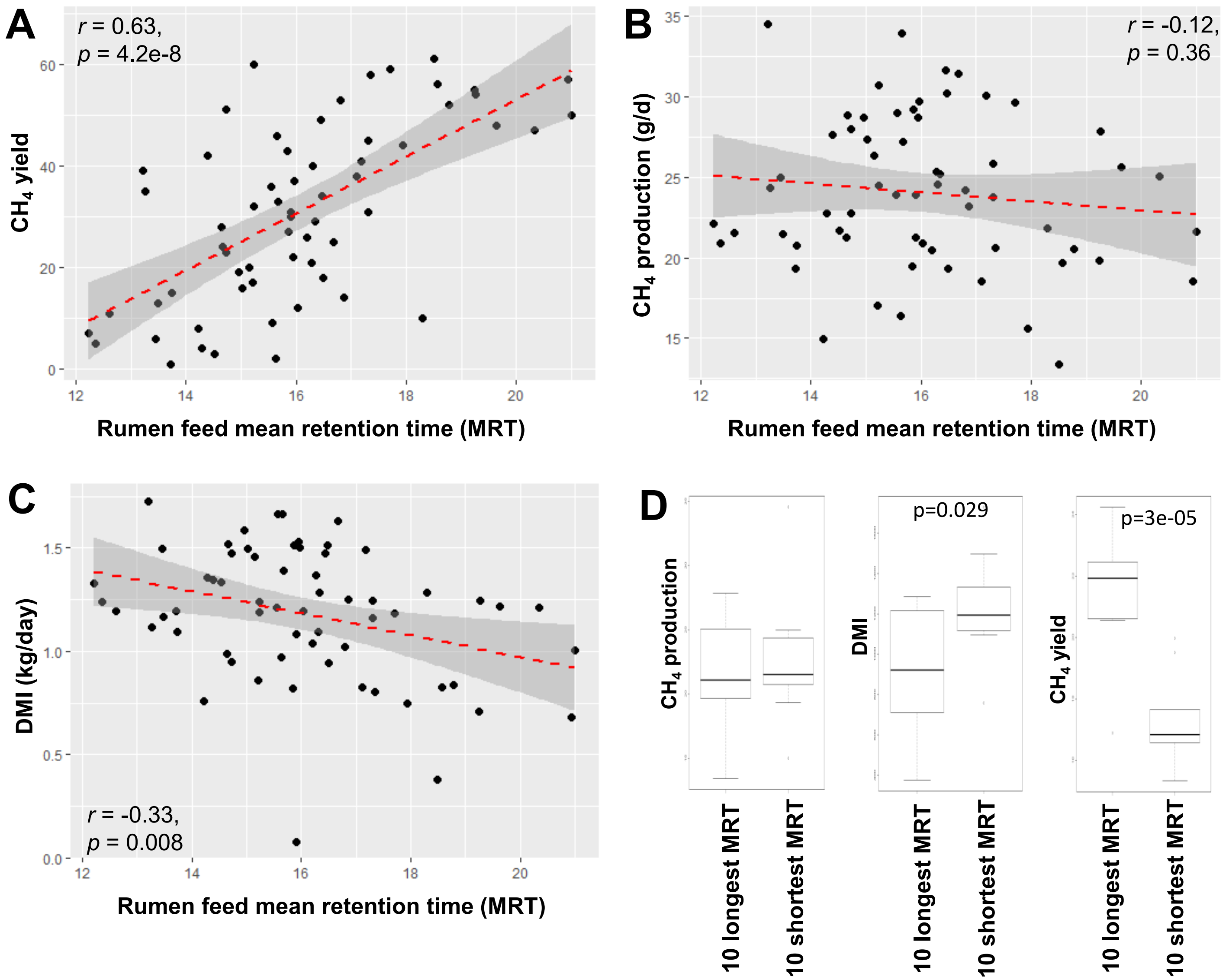

Figure S6. Relationships between CH_4 production, CH_4 yield, dry matter intake (DMI) and mean retention time (MRT) in the Australian CH_4 experiment. Such relationships were shown for all Australian animals (**A-C**) and for top and bottom 10 MRT animals (**D**). MRT unit is hours (h) and the CH_4 yield unit is (g/day/dry matter intake). The CH_4 yield unit is (g/day/dry matter intake), CH4.prod (g/d), DMI (kg/d). Correlation coefficients (*r*) and significance (*p*) were indicated.
