# Supplementary material for: Across-Experiment Transcriptomics of Sheep Rumen Identifies Expression of Lipid/Oxo-Acid Metabolism and Muscle Cell Junction Genes Associated With Variation in Methane-Related Phenotypes
Source: Front Genet. 2018 Aug 20;9:330. doi: 10.3389/fgene.2018.00330 (PMC6109778; doi:10.3389/fgene.2018.00330)
Supplement: FIGURE S7 — Relationships between rumen muscle parameters and CH4 yield and MRT. [file Image_7.pdf]

Figure S7

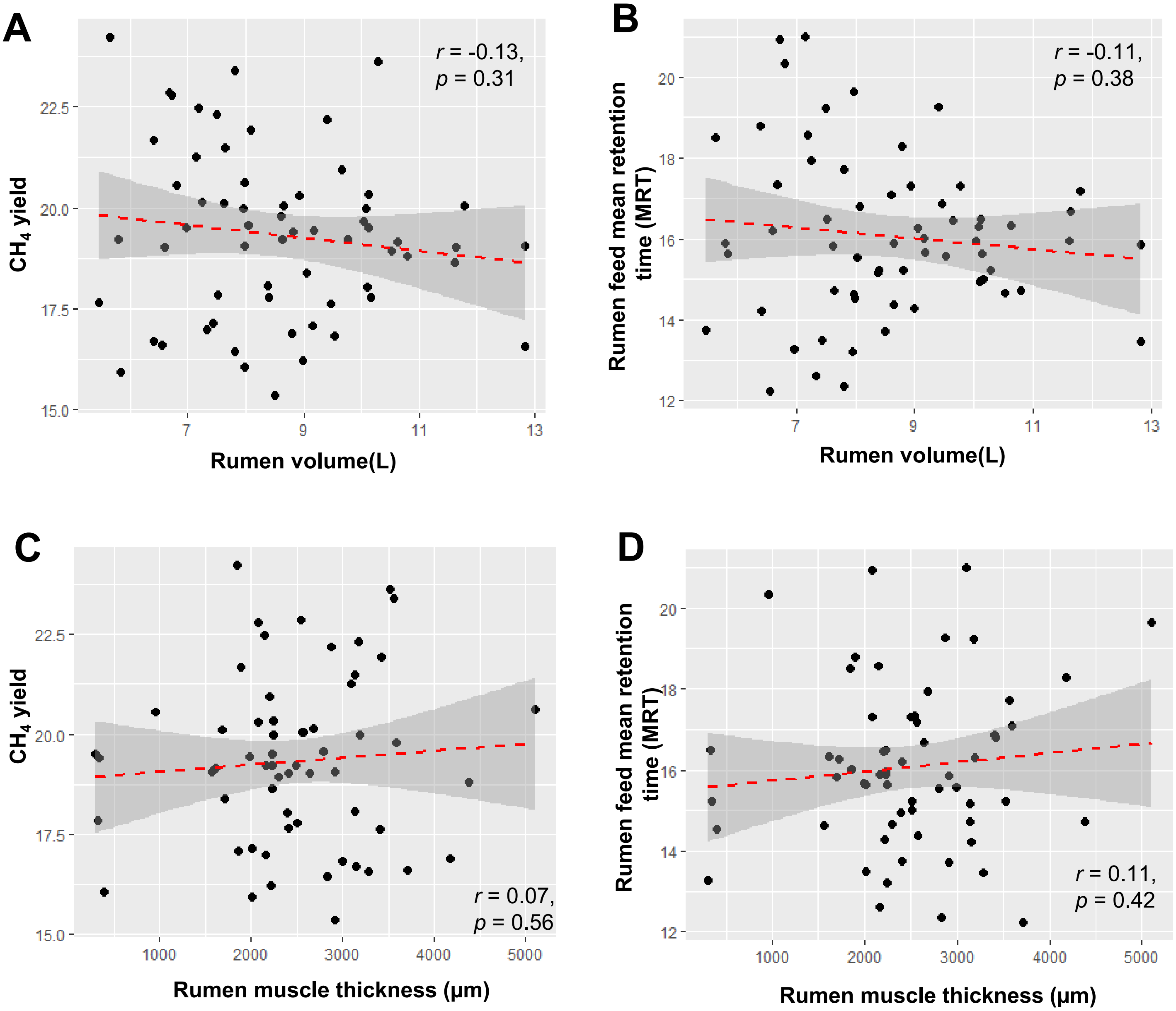

Figure S7. Relationships between rumen muscle parameters and CH_4 yield and mean retention time (MRT) in the Australian experiment. MRT unit is hours (h) and the CH_4 yield unit is (g/day/dry matter intake). Correlation coefficients (*r*) and significance (*p*) were indicated. The measurement of rumen muscle thickness was part of the experiments associated with (Bond et. al. 2017). The whole depth ventral rumen wall tissue was cleaned and the frozen tissue was cryostat sectioned (10 - 12 mm) and stained in haematoxylin solution then rinsed in H_2O. The stained sections were scanned on NanoZoomer 2.0 RS and the muscle depth (mm) was measured using a straight line tool perpendicular from the surface of the outer layer of muscle to the edge of the inner side of the muscle layer.

Bond, J., Cameron, M., Donaldson, A., Austin, K., Harden, S., Robinson, D., et al. (2017). Aspects of digestive function in sheep related to phenotypic variation in methane emissions. Animal Production Science (online version: https://doi.org/10.1071/AN17141).
